# Effect of Indenter Nose Shape and Layer Configuration on the Quasi-Static Perforation Behaviour of Metal–Plastic Laminates

**DOI:** 10.3390/ma15175879

**Published:** 2022-08-25

**Authors:** Mohammad Uddin, Graham Stevens, Daniel Williams

**Affiliations:** 1 UniSA STEM, University of South Australia, Mawson Lakes Campus, Mawson Lakes, SA 5095, Australia; 2 St Vens Pty Ltd., Toowoomba, QLD 4350, Australia; 3 Future Industries Institute, University of South Australia, Mawson Lakes Campus, Mawson Lakes, SA 5095, Australia

**Keywords:** metal–plastic laminates, indenter shape, adhesive, perforation energy capacity, deformation, bonding interfacial failure mechanisms

## Abstract

This study investigated the perforation resistance behaviour of metal–plastic laminates (MPLs) when they are indented by different nose shapes. Aluminium (Al) and HDPE (high-density polyethylene) layers were bonded with a suitable adhesive in an alternative manner to prepare bilayer and trilayer MPL configurations. Quasi-static perforation experiments were performed with hemispherical, conical and blunt indenters. The effects of nose shape, layer configuration and adhesive on the force–deformation profile, perforation resistance capacity and failure mechanisms were evaluated. The results indicate that for a monolithic layer, the blunt indenter showed the highest perforation energy capacity. The conical and blunt indenters facing Al backed by HDPE gave higher perforation energy. The hemispherical indenter facing HDPE backed by Al was found to be more effective in perforation resistance. Trilayer Al–HDPE–Al showed higher perforation resistance than HDPE–Al–HDPE. Circumferential cracking, radial symmetric cracking and shear plugging were the main failure modes for Al under hemispherical, conical and blunt indenters, respectively. The adhesive contributed to an increase in the perforation energy and peak force to failure in laminates. The adhesive was shown to detach from the Al surface after Al fracturing through crack propagation, and this effect was more pronounced when the indenter faced HDPE at the front of the laminate.

## 1. Introduction

In recent decades, there has been a growing interest in using lightweight structures with a specific energy absorption capacity and fatigue resistance in the aerospace, automotive and defence industries when the structures are subjected to impact loading. In this regard, fibre metal laminates (FMLs) composed of a number of thin metal sheets with piles of composite material (prepregs) stacked together have attracted significant attention due to their outstanding impact and fatigue properties [1]. Examples of FMLs include GLARE (glass fibre-reinforced aluminium), CARALL (carbon fibre-reinforced aluminium laminate) and ARALL (aramid fibre-reinforced aluminium laminate) [2]. Despite their success, epoxy matrix cracking and fibre delamination within the composite are a few limitations that have negatively affected the performance of FMLs in targeted applications [3,4]. 

As an alternative, metal plastic laminates (MPLs) are widely used to enhance the impact resistance of lightweight structural components. MPLs are composed by stacking of metal and plastic sheets bonded together with an adhesive. Unlike the time-consuming, complex and expensive composite fabrication process for FMLs, MPLs can be made easily and quickly with commercially available metal and plastic. Recently, due to their superior specific strength, mechanical and corrosion properties, MPLs have been used in manufacturing ship hulls, marine boats, leisure yachts and even chemical tanks, in which the metal provides the overall strength while the plastic layers facilitate energy absorption by plastic stretching and protect the laminates from unwanted impact and corrosive environments (e.g., saline water). For example, in the marine environment, the impact events may include boats or yachts colliding with lighter fishing boats or being hit by external objects or debris during docking. On the other hand, for storage tanks, the impact could be caused by craft tools dropping and hitting the laminate during production or impact by external flying objects in factory setting. All of these impact scenarios are assumed to be very low-velocity impact events for MFLs. In particular, the crashworthiness of a metal–fibre sandwich structure under projectile impact and blast impact has been rigorously investigated using both analytical and numerical approaches [5,6,7].

However, their performance under impact loading with projectiles (indenter) of different nose shapes/geometry has seldom been focused on. In other words, how the plastic or metal layer facing the indenter or the alternating layer configuration affects the deformation and impact resistance is critical to the overall failure of the laminates. 

Recently, Mohangheghian et al. [8] demonstrated that the thickness of the polymer influences the deformation and perforation resistance of metal–plastic laminates when impacted by a blunt indenter. However, the nose shape of the projectile is sensitive to the deformation mechanism, as has been extensively studied for monolithic metal sheets [9]. For example, a blunt indenter causes failure in a monolithic metal by plugging [10], while a hemispherical intender causes it by circumferential fracture [11] and a conical indenter through petal bending [12]. The degree of failure, however, depends on the nose angle and the target material’s properties. Teng et al. [13] studied multilayer metallic targets, and reported that the perforation resistance not only depended on the layer thickness but also on the nose shape and the layer configuration. 

Metal–plastic bilayer laminates were studied for a single indenter shape. For instance, Roland et al. investigated elastomer–steel laminates with a blunt indenter [14], while Radin and Goldsmith studied an aluminium–polycarbonate bilayer impacted by a conical indenter [15]. The low-velocity impact damage of a PMMA–aluminium bilayer bonded with an epoxy adhesive was studied by Liu and Liaw [16], and they found that the damage was highly significant when the thin aluminium layer faced the front of the impactor. Xue et al. [17] studied frictional contact in steel–polyurea bilayer laminates and reported that placing a polyurea backing on the distal face of a steel plate is more effective for a conical projectile and rather less effective for a blunt projectile. The similar effect of the indenter’s nose shape on the deformation in metal–plastic and metal–composite laminates were studied elsewhere in the literature [18,19]. 

In general, numerical modelling (finite element analysis) and experimentation are used to analyse the impact deformation characteristics of the laminates. FEA is a powerful tool to generate results with multitude of design variables in a short period of time, but requires extensive experiments to determine the material properties being fed into the model for accurate predictions [20]. High-velocity impact tests are resource-intensive and time-consuming. Therefore, an attractive alternative option is to reduce experimental overheads to obtain the impact behaviour through the use of simpler, cheaper and more widely adopted quasi-static testing. In literature, quasi-static tests are often used to predict the dynamic deformation characteristics of ductile materials or their fibre composites. It also avoids the problems associated with the filtering or obscuring of important features by force signal oscillations [21]. 

Despite the studies reviewed earlier addressing the nose sensitivity on the impact performance of metal–polymer bilayer laminates, there are few or no reports focusing on the role of the adhesive interface in laminates under the impact of different indenter nose shapes. Further, sandwiched trilayer laminates have not been studied. Therefore, the present study aimed to investigate the performance and failure mode of bilayer and trilayer aluminium–HDPE laminates in quasi-static perforation when subjected to three indenter nose shapes: hemispherical, conical and blunt. In addition to the deformation of the polymer and metal sheets, the role of the adhesive at the interface in the overall failure of the laminates has been studied. The force–displacement gradient, specific perforation energy and peak force to failure of the laminates were discussed and analysed with respect to their monolithic equivalents. 

## 2. Materials and Methods

### 2.1. Materials

In this study, an aluminium alloy (7075-T6) sheet of *h_m_* = 0.5 mm thickness (supplied by Airport Metals Pty Ltd., Melbourne, VIC, Australia) and an HDPE (high-density polyethylene) plastic sheet (black in colour) of *h_p_* = 1.5 mm (supplied by The Plastic Centre Pty Ltd., Melbourne, VIC, Australia) were used to form the laminates. Their key properties are listed in Table 1. The thickness of both the aluminium and HDPE was chosen so that the mass per unit of area of the layer remained constant, which was estimated to be about 1.4 kg·m^−2^. Two commercial adhesives, namely (1) Bostick ISR 70-03 (moisture cure adhesive) (supplied by Bostik Pty Ltd., Melbourne, VIC, Australia) and (2) SoudaSeal 2K hybrid polymer (supplied by Soudal Pty Ltd., Glendenning, NSW, Australia), were used to fabricate the laminates.

### 2.2. Laminate Preparation 

The sample strips of 120 mm × 120 mm were guillotined from the supplied Al 7075-T6 and HDPE plastic sheets. The HDPE surfaces were wiped clean with ethanol on a clean cotton cloth to remove any surface contaminants. A chemical surface treatment [22] was then carried out on one side of the HDPE sample to increase the surface activation energy, which enabled its proper bonding with other surfaces. This was followed by the application of a thin film of Bostik’s Simson ISR 70-03 (Bostik Pty Ltd., Melbourne, VIC, Australia) to the treated surfaces. The application of this adhesive was kept as thin as practical, hand-applied to each sample piece and spread across the substrate surface using a spatula. The ISR 70-03 adhesive was allowed to cure for 2 days. 

To aid further adhesion, the aluminium was sanded with 120 grit sandpaper in a circular motion. Low pressure by hand was applied to provide very light scoring on the surface. Sanded surfaces were wiped free of any contaminants or particles with ethanol on a cotton cloth.

The laminates were constructed by bonding the roughened aluminium and chemically treated HDPE substrates (with the HDPE having the pre-cured layer of Bostik ISR 70-03 adhesive), using elastic Soudaseal 2K (Soudal Pty Ltd., Glendenning, NSW, Australia) adhesive. The adhesive was applied in a thin film to both contacting surfaces of each substrate and then pressed firmly, working from the middle out to each side to remove any excess adhesive and air bubbles. Samples were mechanically clamped for a minimum of 1 h until the adhesive had cured to touch-dry. The above procedure was followed to fabricate bilayer (Al–HDPE) and trilayer sandwiched (Al–HDPE–Al and HDPE–Al–HDPE) laminate samples.

### 2.3. Quasi-Static Perforation Experiments

**Figure 1:**
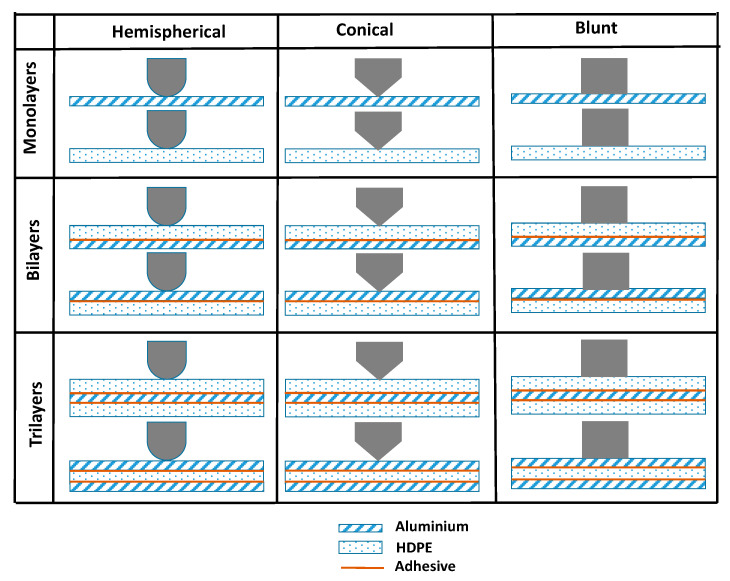
Schematics of the indenter and laminate configurations used in quasi-static perforation tests.

Figure 1 shows a schematic diagram of the laminate configurations, including the monolithic laminates, perforated by three indenters: hemispherical, conical and blunt. For the bilayer laminates, both the aluminium and HDPE surface fronts faced the indenter tip. 

**Figure 2:**
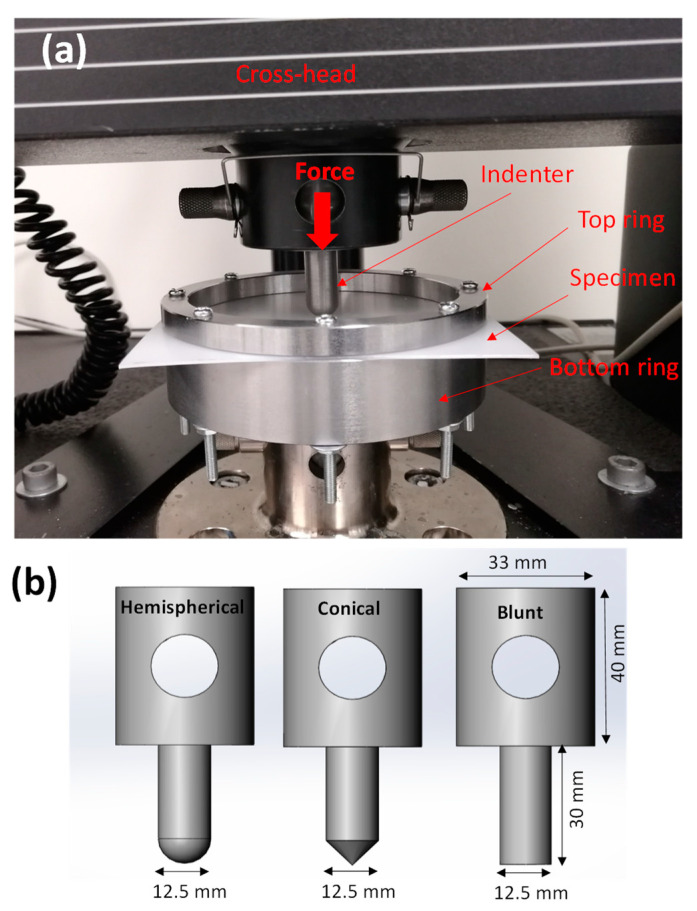
(**a**) Experimental setup for the quasi-static perforation tests and (**b**) the indenters’ geometric dimensions.

Quasi-static perforation experiments were performed on the fabricated laminates with the circular targeted area fully clamped around the edge at a radius Rp = 50 mm. Figure 2a shows the experimental setup. The boundary condition of the laminates was given by a top circular steel ring with an inner diameter of 100 mm clamped by 8 M4 bolts through clearance holes on the laminates sited on the bottom circular steel support plate.

As is shown in Figure 2b, three indenters made of medium carbon steel with unique nose shapes—hemispherical, conical and blunt, each with a diameter of 12.5 (radius Ri = 6.25 mm) mm—were used in the perforation experiments. The ratio of the indenter radius to the laminate radius was Ri/Rp = 0.125. The indenters were machined from mild steel with semi-finish quality and were not assumed to undergo any plastic deformation. 

Perforation experiments were conducted on a material testing machine (Model: Instron 5567, Instron Pty Ltd., Melbourne, VIC, Australia) with a load cell capacity of 30 kN. The indenters were mounted on the load cell, which was attached to the cross-head of the test machine with the specimen and clamping plate beneath. The load cell measured the indentation force while the cross-head displacement gave the indentation distance. A downward cross-head speed of 2 mm/min was used for all quasi-static tests, and the test stop criterion was set to the point when the laminate sample was completely perforated. From the indentation force and displacement data measurements, the specific perforation energy and peak force to failure were estimated to evaluate and compare the energy absorption capacity of the laminates. The specific perforation energy (*E_sp_*) is defined as the ratio between total perforation energy (*E_t_*) and mass per unit of area (*m_l_*) of the laminate. This can be expressed as:

(1) $$m_{l}=\rho_{m}h_{m}+\rho_{p}h_{p}$$

(2) $$E_{sp}=E_{t}/m_{l}$$

where $\rho_{m}$ and $\rho_{p}$ are the density of the metal (aluminium) and the plastic (HDPE), and $h_{m}$ and $h_{p}$ are the thickness of the metal and plastic layers, respectively. 

The performance of the laminates was evaluated with respect to that of the monoliths. For each laminate, the test was repeated three times and their average was considered the final value. 

### 2.4. Failed Specimen Characterisation 

At the end of the tests, the perforated laminates were observed under an optical microscope (Nikon’s stereo microscope: SMZ 745T, Nikon Australia Pty Ltd., Rhodes, NSW, Australia) to analyse the failure mechanism at the entry and exit faces of the laminates. Perforated zones were further sectioned by a hacksaw, and the cross-sections were observed under the same microscope to investigate failure at the interface between the aluminium and HDPE sheets. FTIR tests (Perkin Elmer’s L1600300 Spectrum Two LITA, Perkin Elmer Pty Ltd., Mulgrave, VIC, Australia) were performed to investigate the functional groups of the HDPE and adhesive when bonded together to form the laminate.

## 3. Results

### 3.1. Monolithic Laminates

#### 3.1.1. Force–Displacement Profile

**Figure 3:**
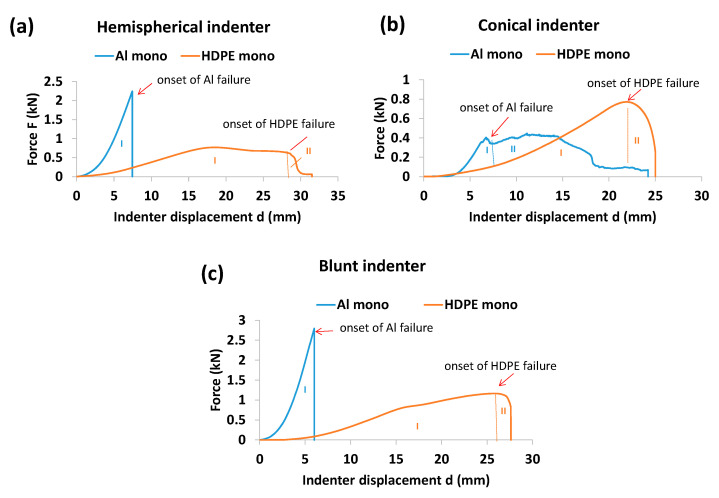
Indenter force and displacement profiles for monolithic aluminium and HDPE when they were perforated by (**a**) hemispherical, (**b**) conical and (**c**) blunt indenters.

Figure 3 shows the quasi-static response of the monolithic aluminium and HDPE subject to indentation by the three indenters. For both materials, the first force peak drop indicates the onset of failure. In other words, this is the point after which the indenter starts to penetrate into the material. Overall, two regions of deformation were observed for the three indenters. Region I indicates the plastic deformation in terms of bending and stretching, reaching the onset point of failure or fracture due to the indenter’s displacement into the material. This is the region where most of energy is supposed to be absorbed by the material. Region II is the further progression of failure in terms of continual cracking or stretching, reaching towards complete perforation. For aluminium, the hemispherical and blunt indenters showed a significant Region I with a clear load drop peak (Figure 3a,c), while the conical indenter showed two obvious regions of deformation (Regions I and II) (Figure 3b). 

It can be seen from Figure 3 that the blunt indenter showed a higher gradient of force with respect to displacement, as compared with the hemispherical and conical indenters. This is due to the larger loading patch or contact area between the indenter tip and the material as the deformation progressed. On the other hand, the hemispherical and blunt indenters revealed higher yielding to the first onset of failure compared with the conical one. HDPE showed significant stretching and elongation until its failure, which may have provided extra energy absorption capacity. 

#### 3.1.2. Perforation Energy and Peak Force to Failure 

**Figure 4:**
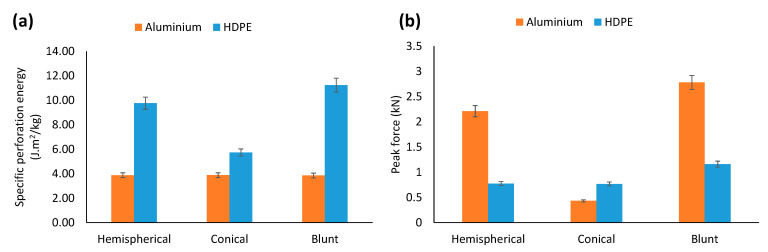
Comparison of the (**a**) specific perforation energy and (**b**) peak force to failure for monolithic aluminium and HDPE under hemispherical, conical and blunt indenters.

Figure 4 shows a comparison of the specific perforation energy and peak force to failure of the monolithic layers for the three indenters. It can be seen from Figure 4a that, for all indenters, due to the nature of the molecular structure, HDPE showed a higher energy absorption capacity than aluminium, and its maximum increase of 191% was noticed for the blunt indenter. 

For aluminium, the indenter’s nose shape had an insignificant influence on the perforation energy. However, for HDPE, the blunt indenter exhibited the highest perforation energy (11 J·m^2^/kg), followed by the hemispherical (9.76 J·m^2^/kg) and conical indenters (5.74 J·m^2^/kg). 

We can also see that for the conical indenter, most of the energy was absorbed by Region II, as opposed to the case for the hemispherical and blunt intenders, where Region I was responsible for most of the energy absorption. On the other hand, for HDPE, Region I was responsible for most of energy absorption under all three indenters. In other words, Region II made an insignificant contribution. 

As can be seen from Figure 4b, due to its high stiffness, aluminium showed higher resistance to yielding when perforated by the hemispherical and blunt indenters than HDPE. However, this was opposite for the conical indenter. Due to its larger contact and loading patch area, the blunt indenter had the highest peak force to first onset of failure, followed by the hemispherical and conical indenters (Figure 3b). This trend remained the same for both the aluminium and HDPE monolayers. Therefore, combining the strength of an aluminium layer and the plastic stretching of a HDPE layer could be used to increase the energy-absorbing capacity of a laminate. 

#### 3.1.3. Monolithic Failure Mechanisms 

**Figure 5:**
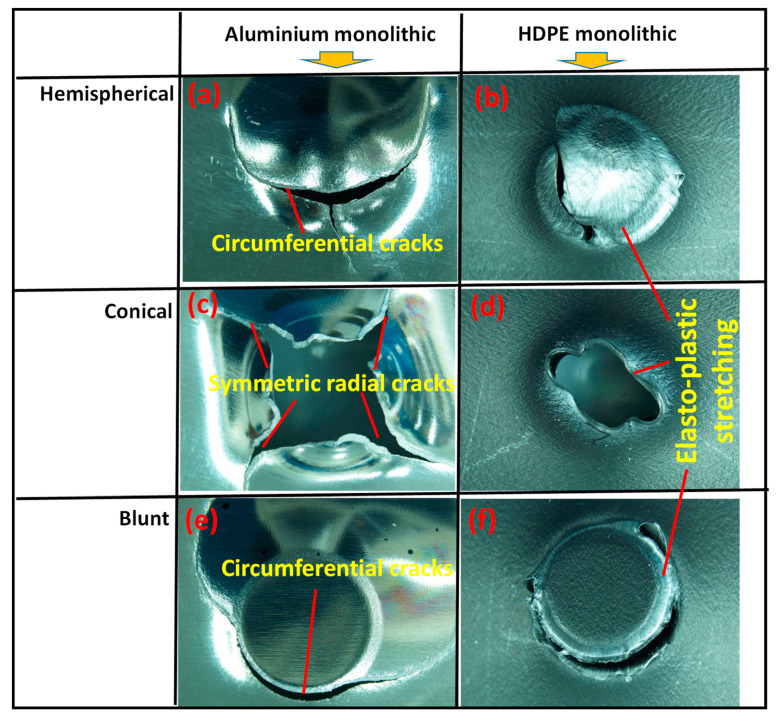
Failure modes of monolithic aluminium (**a**,**c**,**e**) and HDPE (**b**,**d**,**f**) perforated by different indenters. Only the back surfaces of the specimens are shown to illustrate the failure and deformation mechanisms.

Figure 5 shows the failure modes of aluminium and HDPE under the three indenters when observed on the backside or exit of the sheet. For the hemispherical indenter, the failure of aluminium started to occur with a circumferential crack at the indenter tip, and the circular cap was detached due to further displacement of the indenter. A sharp radial crack starting from the tip was observed. Final failure was associated with the propagation of both circumferential and radial cracks, but their contribution to energy absorption was found to be relatively very low, as can be noticed from the force–displacement profile in Figure 3. 

The failure mode for the conical indenter was related to the gradual formation and propagation of four symmetric radial cracks in the aluminium under the tip of the indenter. The combined effect of more gradual bending and cracking would be responsible for the relatively higher energy absorption in Region II for the conical indenter. 

Similar to the hemispherical indenter, for the blunt indenter, the failure started from a circumferential crack, causing detachment of a radial cap, similar to shear plugging through further crack propagation. Relatively similar failure modes were noticed for HDPE when subjected to three indenters, but instead of cracks, significant elastic–plastic stretching was the dominant mechanism of deformation until perforation. 

### 3.2. Bilayer Laminates

#### 3.2.1. Force–Displacement Profiles

**Figure 6:**
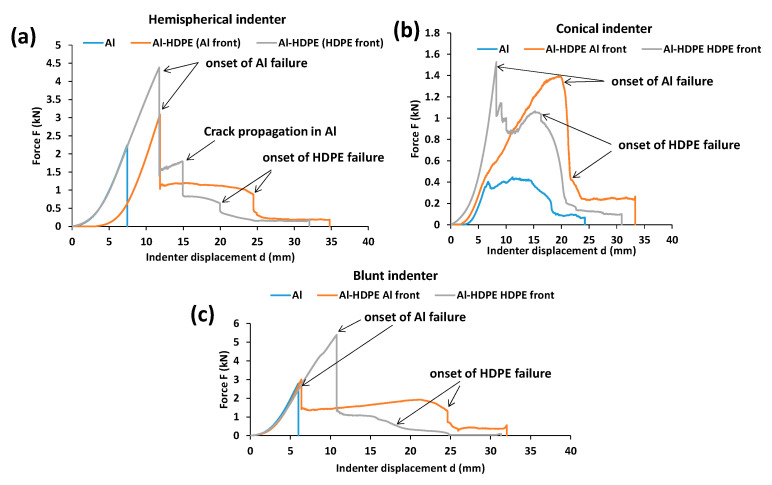
Quasi-static force-displacement profile of bilayer laminates under (**a**) hemispherical, (**b**) conical and (**c**) blunt indenters.

Bilayer laminates of Al–HDPE were bonded with the adhesives (as explained in Section 2) and tested under the three indenters. The tests involved each indenter facing both the aluminium and HDPE separately to determine their quasi-static responses for different combinations of the backing material used in the laminates. Figure 6 shows the indenter force–displacement profiles of the bilayer laminates. 

In the case of the hemispherical indenter (Figure 6a), the force–displacement profile of the Al–HDPE laminate, whether the indenter faced either the aluminium (Al) or the HDPE, remained same in Region I until it reached the first peak load drop where the aluminium failed due to fracture. In other words, the quasi-static response for the bilayer laminate in Region I follows a similar response to monolithic aluminium (Figure 2a). Region II was predominantly associated with plastic stretching of the HDPE together with the adhesive interface until complete perforation through the HDPE at the second peak load drop. However, in case of the indenter facing the HDPE surface, an intermediate load peak drop was noticed. This could be attributed to the propagation of cracks within the aluminium until the HDPE was perforated.

For the conical indenter facing the aluminium surface, the force–displacement gradient was almost same as that of monolithic aluminium (Figure 6b). When HDPE was at the front facing the indenter, the gradient was very high until the first peak load drop, followed by a gradual drop in the load for sustained displacement until reaching the second peak load drop. Larger deformation regions between the first and second peak loads would be influenced by significant plastic bending and stretching of the HDPE. 

In the case of the blunt indenter, there was a negligible change in the force–displacement gradient regardless of the indenter facing either the Al or HDPE (Figure 6c). However, when the indenter faced aluminium, the Al failed at relatively a smaller displacement, after which, gradual bending and stretching of the softer HDPE plastic was the dominant mechanism of deformation until the HDPE was perforated. On the other hand, higher resistance to the first peak load drop was observed when HDPE was at the front. This was due to the larger contact patch between the plastic and the indenter tip, backed by aluminium, hence delaying the first onset of failure in aluminium, followed by gradual perforation through the HDPE. In this case, most of energy is thus supposed to be absorbed by the first region of deformation. 

Thus, it is clear that having HDPE at the back of the laminate increases the strength to failure of the laminate by delaying the fracture failure of aluminium. In other words, stretching in the HDPE causes an increase in the contact area between the indenter tip and the laminate, and lowers the contact pressure and failure propensity. 

#### 3.2.2. Perforation Energy and Peak Force to Failure

**Figure 7:**
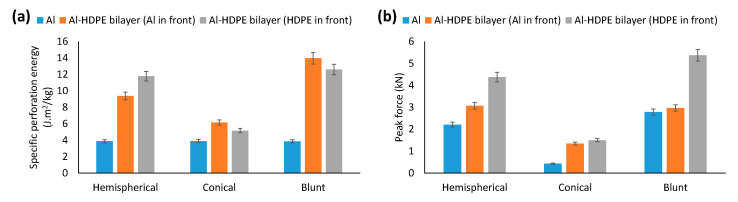
Comparison of (**a**) perforation energy and (**b**) peak force for Al–HDPE bilayers under hemispherical, conical and blunt indenters.

Figure 7 shows a comparison of the specific perforation energy and peak force to failure for Al–HDPE bilayers. Compared with monolithic aluminium, adding a HDPE layer in the laminate increased the perforation energy and resistance to failure. For the hemispherical indenter facing HDPE at the front, the laminate showed higher perforation energy (11.78 J·m^2^/kg) than when the indenter faced aluminium at the front (9.37 J·m^2^/kg) (Figure 7a), which accounted for a 26% increase. On the other hand, this is opposite to the case for the conical and blunt indenters, in which, the indenter facing aluminium exhibited higher perforation energy capacity, which accounted for 19% and 11% increases for the conical and blunt indenters, respectively. This result is quite consistent with their force-displacement profiles presented in Figure 6. Thus, aluminium backed by HDPE is preferred for the conical and blunt indenter to increase the perforation energy, and HDPE at the front is desired for the hemispherical indenter. 

As can be seen from Figure 7b, for the three indenters, Al–HDPE laminate with the HDPE facing the blunt indenter showed the highest peak force (5.4 kN) (i.e., resistance to the first onset of aluminium failure), while both the hemispherical and conical indenters showed equal resistance (about 4.4 kN). 

#### 3.2.3. Bilayer Failure Mechanisms

**Figure 8:**
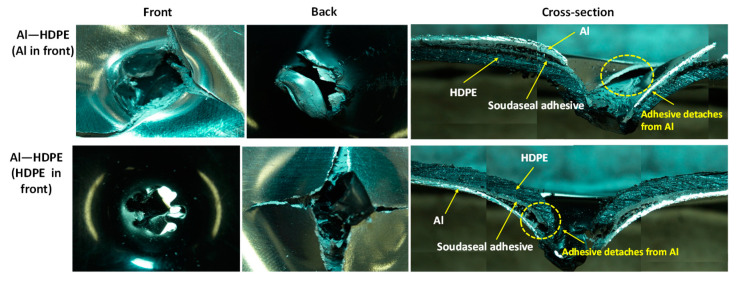
Failure modes of bilayer laminates under the hemispherical indenter.

For the hemispherical indenter, the failure modes at the front and back of the bilayer followed their monolithic equivalents, regardless of whether aluminium or HDPE faced the indenter tip (Figure 8). Aluminium failed through initiating and propagating four symmetric regular cracks, while HDPE stretches through plastic bending and is perforated with a clean round hole. The cross-sectional views shown in Figure 8 provide a detailed illustration of the inner workings and interfacial failures involving aluminium, HDPE and the adhesive of the laminate.

When aluminium or HDPE faced the indenter, the Soudaseal adhesive easily separated from aluminium surface at the perforation zone, while the adhesive stuck well to the HDPE surface. This was due to the propagation of cracks through the aluminium, thus physically disconnecting the adhesive from the metal, and due to the mismatch in plastic bending between the strong aluminium and the softer HDPE.

**Figure 9:**
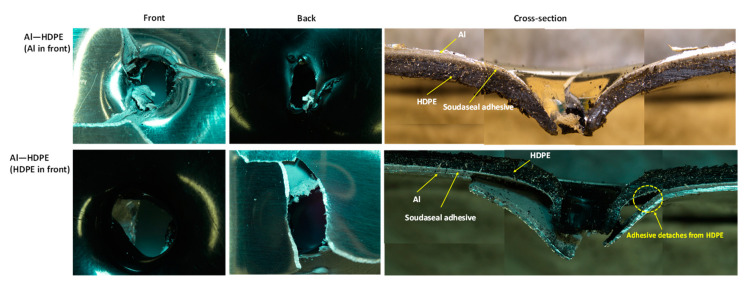
Failure modes of bilayer laminates under the conical indenter.

As shown in Figure 9, for the conical indenter, aluminium failed through three symmetric cracks when the indenter tip faced aluminium at the front, but spiral-shaped cracks in aluminium were noticed when the indenter faced HDPE. Clean perforations were observed for HDPE. As shown in the cross-sectional view of the failed samples, when the indenter faced aluminium backed by HDPE, there was no noticeable separation between the adhesive and the aluminium. The adhesive was rather stretched along with the bottom HDPE layer. This would be the reason why Al–HDPE with Al in front facing the conical indenter showed a greater perforation energy capacity. However, the opposite was observed when HDPE backed by Al faced the indenter, and, due to crack propagation, the aluminium separated from the adhesive easily, and HDPE stretching contributed to further perforation energy. 

**Figure 10:**
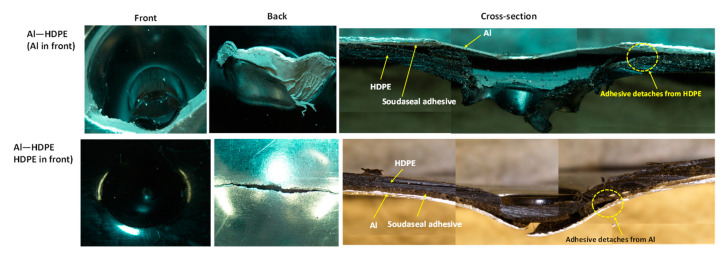
Failure modes of bilayer laminates under the blunt indenter.

As shown in Figure 10, for the blunt indenter, due to the large contact patch between the indenter tip and the aluminium or HDPE sheet, the failure started at the periphery of the indenter. The cross-sectional view shows that when aluminium faced the indenter, it failed through cut-out at the periphery, and the adhesive detached from the aluminium around the failure zones. In the meantime, the HDPE plastically stretched and provided extra support, thus delaying the perforation. On the other hand, when HDPE faced the indenter, the combined effect of the HDPE, adhesive and aluminium deformations contributed to energy absorption, until the aluminium failed. The adhesive stuck relatively well to the HDPE and aluminium beyond the periphery of the failure zone.

### 3.3. Trilayer Sandwiched Laminates 

#### 3.3.1. Force–Displacement Profiles

**Figure 11:**
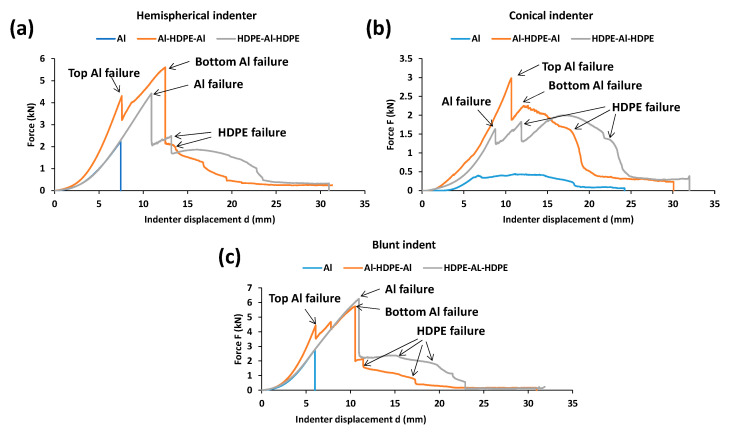
Quasi-static force and displacement profiles of trilayer laminates under the (**a**) hemispherical, (**b**) conical and (**c**) blunt indenters.

As shown in Figure 11a, for the hemispherical indenter, the fundamental force–displacement profile for sandwiched trilayer laminates (Al–HDPE–Al and HDPE–Al–HDPE) followed that of their monolithic equivalents. Two peaks for the onset of Al failure after a sharp gradient of force were observed for Al–HDPE–Al, in which the first peak represents the fracture of the top Al layer and the second peak indicates the fracture of the bottom Al layer. However, the second peak appears at a higher force, which might be due to the further strengthening of the bottom aluminium layer. As the indenter tip pushes with a large contact area with the laminate, the bottom aluminium layer undergoes significant plastic deformation. This behaviour was not noticed when the aluminium was placed in between two HDPE layers. The highest peak load represents the failure of the Al layer, followed by a lower second peak due to the failure in the HDPE layers. 

As shown in Figure 11b, for the conical indenter, the force–displacement profile is opposite to that of the hemispherical indenter. In other words, two ascending load peaks resembling a reasonable work-hardening effect was noticed for HDPE–Al–HDPE. A similar effect was, however, observed for Al–HDPE–Al under the hemispherical indenter. 

For the blunt indenter (Figure 11c), the Al–HDPE–Al and HDPE–Al–HDPE laminates had a similar force-displacement profile to those with the hemispherical indenter. However, the peak forces for the blunt indenter are relatively higher than that for the hemispherical one. This would allow the laminates to absorb greater perforation energy when subjected to the blunt indenter.

#### 3.3.2. Specific Perforation Energy and Peak Force to Failure 

**Figure 12:**
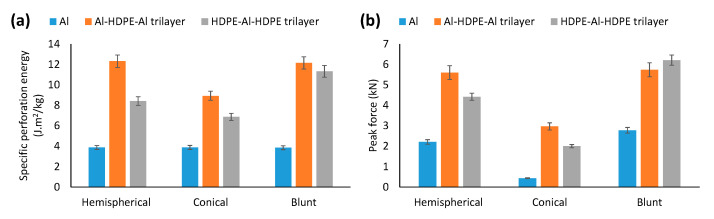
Comparison of (**a**) perforation energy and (**b**) peak force to failure for Al–HDPE–Al and HDPE–Al–HDPE trilayers under hemispherical, conical and blunt indenters.

As shown in Figure 12, regardless of the indenter type, Al–HPDE–Al showed greater perforation energy than HDPE–Al–HDPE. A HDPE layer sandwiched by two aluminium layers would be preferred as opposed to two HDPE layers. The hemispherical and blunt indenters showed almost the same perforation energy, while the conical indenter showed the lowest perforation energy. A similar trend for peak force to failure was noticed for Al–HDPE–Al and HDPE–Al–HDPE laminates compared across the three indenters. This result is consistent with the force–displacement behaviour of the indenters.

#### 3.3.3. Trilayer Failure Mechanisms 

**Figure 13:**
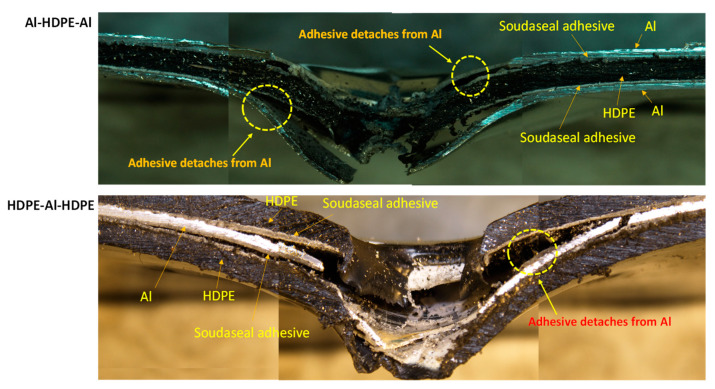
Cross-sectional view of the failure modes of tri-layer laminates under the hemispherical indenter.

As shown in Figure 13, for the hemispherical indenter, the failure mode of the trilayer laminates followed a similar trend and mechanism to the bilayer laminates. After the onset of aluminium fracturing, the aluminium severely detached from the adhesive when the aluminium was not supported by HDPE. This was observed in Al–HDPE–Al. However, when aluminium was backed by HDPE (in the case of HDPE–Al–HDPE), plastic stretching of the HDPE layers slowed down the propagation of the cracks in aluminium, and thus, higher adhesion between the aluminium and HDPE layers was retained.

**Figure 14:**
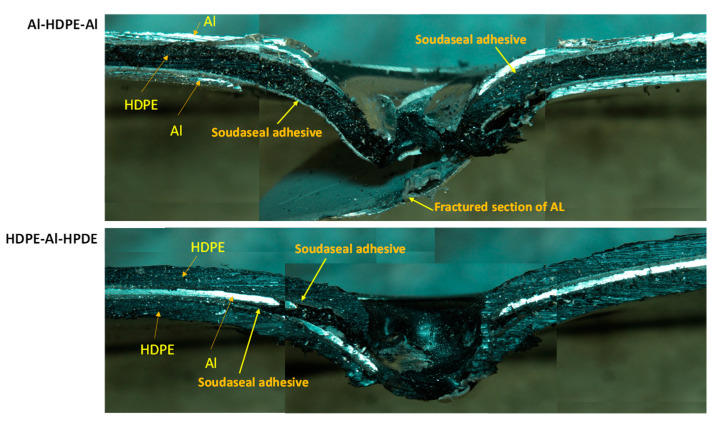
Cross-sectional view of the failure modes of tri-layer laminates under the conical indenter.

Because of the sharp and pointy nose shape of the conical indenter, severe fractures in aluminium were observed for Al–HDPE–Al, as shown in Figure 14. Relatively large plastic bending of the aluminium and stretching of the HDPE along the depth of penetration were observed. This was due to significant frictional resistance between the indenter tip and the laminate until its perforation. However, the indenter facing HDPE at the front caused early penetration into the softer HDPE, while the middle aluminium layer was little supported by the bottom HDPE. 

**Figure 15:**
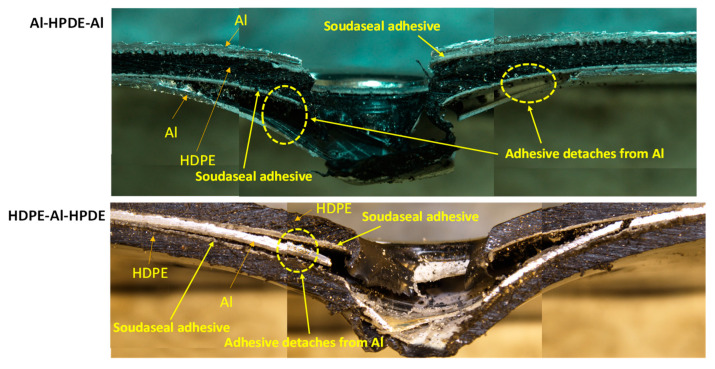
Cross-sectional view of the failure modes of tri-layer laminates under the blunt indenter.

For the blunt indenter (Figure 15), both aluminium and HDPE were subjected to severe fracturing and plugging out around the periphery of the indenter tip, causing a sharp perforation at the top and intermediate layers, while the bottom layer was pushed down until complete perforation throughout. Relatively less plastic bending and stretching in the aluminium and HDPE were observed. The adhesive detached from the aluminium layer right after the onset and propagation of the cracks, causing separation between the aluminium and HDPE layers.

### 3.4. Comparison of the Perforation Energy without Adhesive

**Figure 16:**
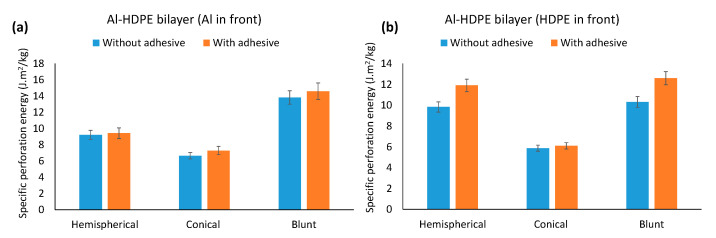
Comparison of perforation energy with and without adhesive for (**a**) when Al faced the indenter and (**b**) when HDPE faced the indenter in Al–HDPE bilayers.

Laminates without adhesives were perforated by the three indenters to investigate the role of the adhesive on the perforation energy of the laminates. As can be seen from Figure 16, for the bilayers, regardless of whether the indenter faced either aluminium or HDPE, the adhesive increased the perforation energy. As shown in Figure 16a, the increase in perforation energy was marginal (2–9.5%) when the indenters faced aluminium. However, this increase with the indenters facing HDPE in the front was significantly larger, as can be noticed in Figure 16b. The increase due to adhesive was 21% and 26% for the hemispherical and blunt indenters, respectively. These results clearly outline that adhesive bonds better with the HDPE surface than with aluminium, and the initial plastic stretching of the HDPE and adhesive layers delay fracture failure in the bottom aluminium layer. This also means that a laminate with HDPE facing the indenter would be preferred to leverage the increased perforation energy capacity. 

**Figure 17:**
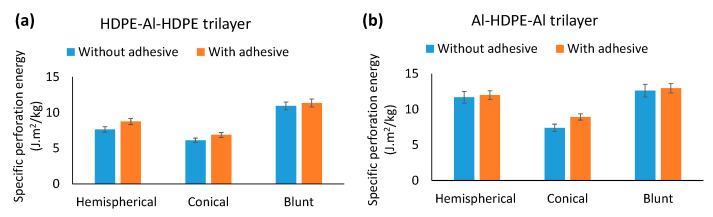
Comparison of perforation energy with and without adhesive for (**a**) Al–HDPE–Al and (**b**) HDPE–Al–HDPE trilayers.

A similar trend for the trilayers (Al–HDPE–Al and HDPE–Al–HDPE) was noticed (Figure 17), but the effect of the adhesive was minimal. This indicates that an adhesive layer with elasto-plastic properties can contribute to the perforation energy. However, it appears that its effect is more prominent and beneficial in the case of bilayer laminates.

In addition, in a comparison of the perforation energy between bilayers and trilayers under the three indenters (Figure 16 and Figure 17), it appears that having an additional HDPE layer in a trilayer HDPE–Al–HDPE laminate did not increase the perforation energy. However, the benefit was clearly noticed when an extra Al layer is added in the trilayer Al–HPDE–Al laminate. 

### 3.5. FTIR Spectra of Laminates with Adhesive

**Figure 18:**
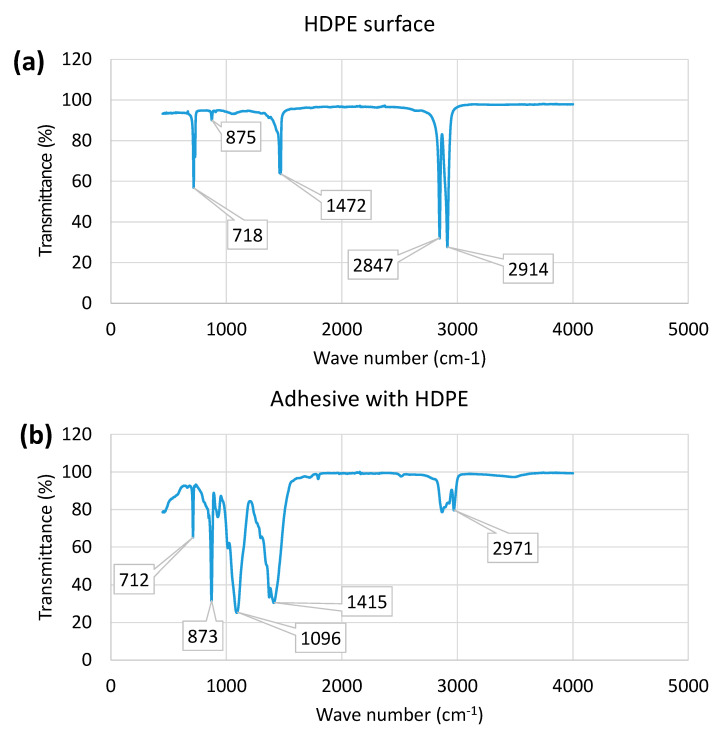
FTIR spectra of the HDPE and adhesive used in the laminates.

Figure 18 shows FTIR spectra of the treated HDPE and Soudaseal adhesive used for bonding in the laminates. As can be seen in Figure 18a, HDPE shows standard polyethylene-based functional groups, which can be identified at four critical peaks. The first peak at ~718 cm^−1^ is associated with CH_2_ rocking, which can be ascribed to the crystallinity of polyethylene. The second sharp peak at ~1472 cm^−1^ is associated with asymmetric CH_2_ deformation vibration of the methylene group. Two closely co-located intense peaks at ~2847 cm^−1^ are associated with symmetrical CH_2_ stretching, and that at ~2914 cm^−1^ is associated with asymmetric CH_2_ stretching of the methylene group. A relatively small peak at ~875 cm^−1^ is associated with the presence of C_2_O_2_ peroxides because of the chemical treatment of HDPE prior to bonding. 

On the other hand, the adhesive on the HDPE’s surface show four significant peaks at lower-order wave numbers (Figure 18b). An additional new peak at ~1096 which is associated with C-C stretching vibrations was noticed when compared with HDPE only. The peak at ~873 is quite intense, which indicates the strong presence of C_2_O_2_ peroxides within the adhesive. This means that prior chemical treatment helps bond the adhesive and HDPE. The peaks at ~1415 and 2971 are associated with CH_2_ scissoring and symmetric CH_2_ stretching vibrations of methylene, respectively, which are present in the hybrid polymer-based adhesive (Soudaseal 2k) used in laminates. The FTIR spectra thus clearly confirm the relevant functional groups in the HDPE, the chemical treatment and the adhesive used, which contributed to the bonding of the laminates. 

## 4. Discussion 

In this study, we investigated the effects of indenter nose shape, configuration and adhesive on the quasi-static perforation resistance of metal–plastic laminates. Monolithic tests showed that the low-strength and highly ductile HDPE had higher specific perforation resistance than high-strength aluminium when tested under the three indenters. As the indenter penetrates, the HDPE undergoes significant plastic stretching, and hence the contact area patch between the indenter and plastic increases, with a prominent effect for the blunt indenter, as opposed to the hemispherical and the conical intenders. While most of the perforation energy is absorbed via elasto-plastic deformation, the key observation is that the metal layer fails through fracturing at the indenter tip: cracking and shear plugging. The finding is quite consistent with the observations reported in [8]. 

We aimed to demonstrate how an HDPE layer can further increase the perforation energy when bonded with an aluminium layer by an adhesive. In other words, what is the underlying reason that defines the deformation characteristics of two combined layers? Our results clearly indicate that an additional HDPE layer further increased the perforation energy of Al–HDPE laminates, compared with monolithic aluminium. When HDPE is at front of the hemispherical indenter, high plastic stretching of plastic, i.e., dishing out, delays the fracture onset of aluminium. In other words, the bottom aluminium undergoes greater plastic deformation, thus enabling higher perforation energy. For the conical and blunt indenters, it was the opposite, i.e., the perforation energy decreased. This could be due to the fact that the sharp, pointy conical indenter caused early penetration into the soft HDPE at the top first, followed by plastic deformation into the bottom layer of aluminium. Similarly, the edge of the blunt indenter causes circumferential damage to the HDPE. Thus, the aluminium rather contributes more to the overall resistance, instead of the HDPE layer. On the other hand, the effects of nose shape on perforation resistance for polymer–metal or metal–fibre composite laminates have been studied by other researchers [18,19], reporting that having softer material facing the indenter increases the perforation resistance or energy absorption capacity. 

Xu et al. [17] reported that a polyurea (PU) polymer layer backing a steel plate can contribute to the increase in energy absorption under high-velocity impact be conical and blunt indenters. The plastic layer enables an increase in energy dissipation in the steel while storing the dissipated energy in the plastic via large stretching. Thus, it is clear that the findings from our quasi-static perforation tests are aligned with those of impact experiments, and the indenters’ nose shape affected the deformation and perforation resistance. Impact-induced glass transition causing an increase in the strength of plastic has been reported in the literature [8,23]. However, in our study, this may not be the case, as we performed quasi-static perforation tests where the strain rate change effect was assumed to be negligible. For a metal–plastic (HDPE) bilayer, Mohagheghian et al. [8] further confirmed this by a comparative analysis of both impact and quasi-static perforation experiments.

Our trilayer results show that when HDPE is sandwiched with two aluminium layers, the hemispherical and conical indenters show an increase in perforation energy, compared with the bilayer (Al at front), while the blunt indenter decreases the perforation energy. However, as reported by Xu et al. [17], there is no benefit when PU plastic is sandwiched with steel layers under the conical and blunt nose shapes. In their experiments, they used relatively thicker steel plates which absorbed most of the perforation energy as the deformation of the PU plastic was constrained by strong metal layers. On the other hand, in our case, aluminium and HDPE are relatively thin, which might have contributed to the higher perforation energy. However, aluminium sandwiched with two HDPE layers did not increase the absorption energy, and hence this may not be a preferred option. 

We demonstrated that a thin adhesive layer between the metal and plastic layers marginally contributed to better bonding and higher perforation energy for bilayers, compared with the laminates with frictional contact (Figure 16 and Figure 17). This might be due to the fact that the adhesive, as an extra polymer layer, retards the onset of necking of the metal layer, and thus increasing energy dissipation and delaying the fracture failure in the metal. However, the significance of neck retardation depends on the quality of the adhesive bonding and the incremental stiffness of the plastic layer. It can, however, be assumed that chemical surface pretreatment of HDPE may have contributed to improved bonding of the laminates studied here, which was confirmed by the FTIR results. The effect of neck retardation due to adhesive was also reported in Xu and Hutchinson [24] for metal–elastomer laminates under biaxial stretching. However, the true contribution of neck retardation in quasi-static perforation under different indenter nose shapes is a matter for further investigation. 

Note that in this study, as a first step, we used the quasi-static indentation approach to understand the deformation and perforation resistance of the laminates under different indenter nose shapes. However, the deformation characteristics might be slightly different when the laminates are subjected to dynamic impact. Though the magnitude of force-displacement and perforation energy capacity will change, the conclusions of this investigation are expected to remain the same. The use of static indentation to predict the dynamic impact for relatively thinner composite laminates was justified by other researchers, suggesting that static tests are adequate for determining the initial impact and onset of damage in a metal or plastic layer through the adhesive interface [21]. Having said that, it is worth investigating the true laminate performance under high-speed impact, which will be the scope of our future work.

## 5. Conclusions

In this study, we reported the quasi-static perforation performance of monolithic, bilayer and trilayer laminates composed of aluminium (Al) and HDPE layers under hemispherical, conical and blunt indenters. For the range of laminate configurations considered here, the following major conclusions can be drawn out from this work.

- For monolithic laminates, the blunt indenter showed the greatest perforation energy capacity for both Al and HDPE, followed by the hemispherical and conical indenters. In particular, HDPE showed a 191% increase in perforation energy compared with Al with the blunt indenter. Circumferential cracking, radial symmetric cracking and shear plugging were the main failure modes for Al in the perforation loading under the hemispherical, conical and blunt indenters, respectively.
- In Al–HDPE bilayer laminates, for the hemispherical indenter, having HDPE facing the indenter tip was more effective for perforation resistance, showing a ~26% increase compared with the case when Al faced the indenter. On the other hand, it was the opposite for the conical and blunt indenters, in which having Al facing the indenter produced higher perforation energy, which accounted for increases of about 19% and 11% with the conical and blunt indenters. The deformation and failure mechanism of the laminates apparently matched that of their monolithic equivalents.
- For all indenters, the adhesive at the interface of the bilayers detached from the Al surface after fracture of the Al through crack propagation. Detachment of the adhesive was more severe with Al as a backing, i.e., when the indenter faced HDPE. This was quite noticeable for the conical indenter. On the other hand, the adhesive stuck well to the Al and provided extra support through plastic stretching until the perforation.
- In trilayer laminates, Al–HDPE–Al showed higher perforation resistance than HDPE–Al–HDPE. The additional HDPE layer in the trilayer HDPE–Al–HDPE laminate did not boost the perforation resistance beyond the bilayer Al–HDPE. The hemispherical and blunt indenters showed almost the same perforation energy, while the conical indenter had the lowest. This resembled the performance of their monolithic equivalents. The failure mode of the tri-layer laminates followed a similar trend and mechanism to the bilayer laminates.
- Confirmed by the FTIR data, the hybrid polymer-based adhesive used for bonding contributed very marginally to the increase of the perforation energy and peak force to failure in laminates. The increase was found to be more prominent for bilayers as opposed to trilayers. Neck retardation due to the adhesive might be the mechanism of the higher perforation energy.

## Figures and Tables

**Table 1:** Key specifications of the materials used in this study.

Material	Product Name	Thickness (mm)	Density (kg·m^−3^)	Young’s Modulus (GPa)	Yield Strength (MPa)	Nominal Failure Strain
Aluminium 7075-T6	AMS 4049	0.5	2810	72	503	0.11
HDPE	HDPE	1.5	960	1.2	26	13.5

## Data Availability

Not applicable.
